# Primary synovial sarcoma of the thyroid gland: a CARE compliant case report and literature review

**DOI:** 10.3389/fmed.2023.1158334

**Published:** 2023-05-10

**Authors:** Chutong Ren, Yashan Li, Jiangsheng Huang, Sushun Liu, Zhexu Cao, Qin Jiang, Xiang Lin, Fei Ye, Yi Gong

**Affiliations:** ^1^Department of General Surgery, The Second Xiangya Hospital, Central South University, Changsha, Hunan, China; ^2^Department of General Surgery, Huaihua Second People's Hospital, Huaihua, Hunan, China

**Keywords:** primary synovial sarcoma of the thyroid gland, SS18 gene, perioperative evaluation, case report, literature review

## Abstract

**Rationale:**

Synovial sarcoma is a subtype of soft tissue sarcoma. Synovial sarcoma in the head and neck region is relatively unusual. Primary synovial sarcoma of the thyroid gland (PSST) is first reported in 2003 by Inako Kikuchi. PSST is extremely rare with only 15 cases documented globally. PSST shows rapid disease progression and a relatively poor prognosis. However, diagnosis and therapy are challenging for clinical surgeons. In this article, we reported the 16th PSST case and reviewed the PSST cases globally for further clinical application.

**Patient concerns:**

The patient was referred to us because of gradually worsened dyspnea and dysphagia for 20 days. Physical examination showed a 5 × 4 cm mass with a clear boundary and good mobility. Contrast-enhanced ultrasonography (CEUS) and computed tomography (CT) showed a mass in the isthmus of the thyroid gland. The imageology diagnosis tends to be a benign thyroid nodule.

**Diagnosis:**

After surgery, histopathology, immunohistochemistry, and fluorescence, *in situ* hybridization indicated the mass to be primary synovial sarcoma of the thyroid gland with no local and distant metastasis.

**Interventions:**

The patient underwent total thyroidectomy and dissected the lymph nodes in the central compartment. This patient received postoperative chemotherapy (a combination of ifosfamide and epirubicin for five cycles). Patients tolerated chemotherapy well. No recurrence was found during the 9-month follow-up.

**Lessons:**

Although PSST is an extremely rare disease, we should raise our awareness when we encounter a rapidly growing, cystic-solid mixed thyroid mass with neck compression symptoms to avoid misdiagnosis. Intraoperatively, surgeons should refine surgical procedures to avoid capsular rupture and tumor local implantation metastasis. Intraoperative frozen section pathology is necessary sometimes, especially when the diagnosis could not be established before surgery.

## Introduction

Synovial sarcoma is a kind of histological subtype of soft tissue sarcoma, making up 8–10% of all soft tissue sarcomas. It is a high-grade malignant neoplasm of mesenchymal pluripotent cells, characterized by a specific t(X;18) (p11.2; q11.2) translocation leading to the generation of SYT-SSX fusion gene transcripts (1, 2). According to different ultra structures, synovial sarcoma was divided into monophasic patterns and classic biphasic patterns (2). Synovial sarcoma in the head and neck region is relatively unusual, and primary synovial sarcoma in the thyroid gland is extremely rare with only 15 cases documented globally (3–13). Due to the rarity of the disease, challenges exist in clinical practice from diagnosis to follow-up. In the study, we report a case of a middle-aged female who was diagnosed with thyroid synovial sarcoma post-surgery. This is the 16th PSST case reported. For deep investigation, the clinical characteristics of 15 previously reported cases were also carefully summarized. Because of a lower survival rate (5-year survival rate of ~60%) than differentiated thyroid cancer (14), we hope our case report and literature review could arise awareness of thyroid synovial sarcoma and improve the diagnosis and treatment of this disease.

## Case presentation

**Figure 1:**
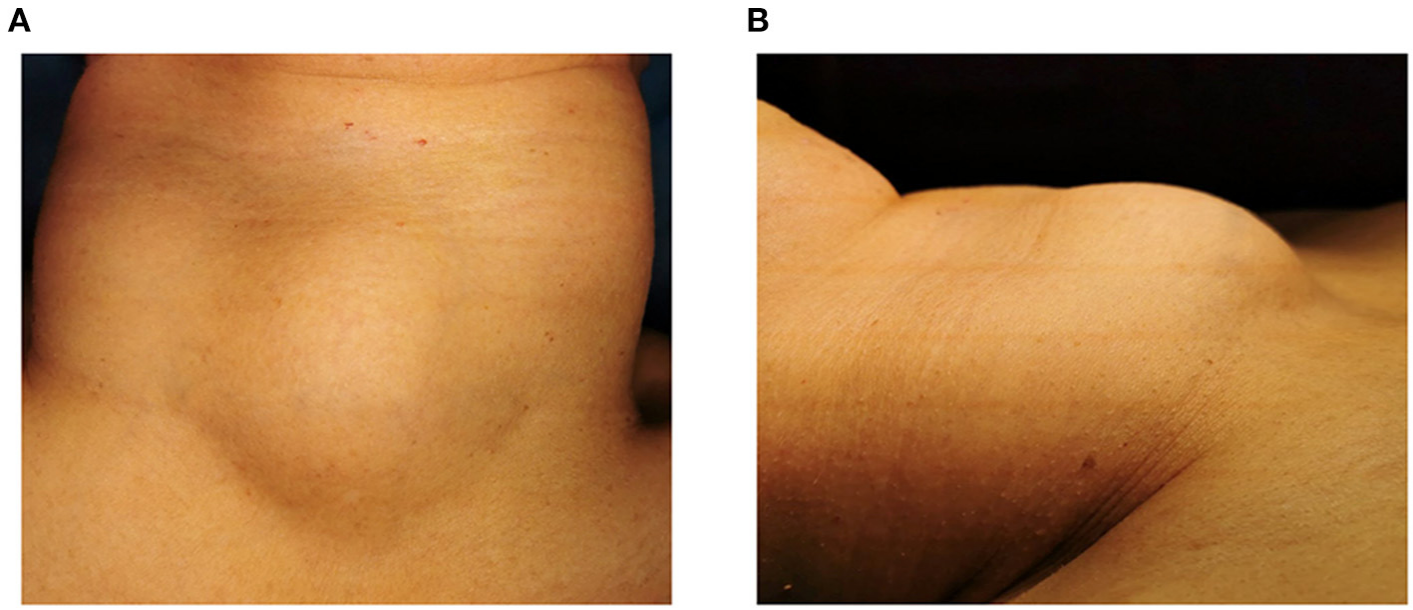
Preoperative photo of the tumor anteriorly **(A)** and laterally **(B)**.

A 59-year-old female patient was admitted to our hospital in June 2022. Her chief complaints were dyspnea and dysphagia for 20 days. She felt dyspnea 20 days before, and the discomfort gradually worsened accompanied by cough and dysphagia. The physical examination showed a palpable hard mass in the center of the neck. The size of the mass was 5 × 4 cm (Figure 1). The boundary of the mass is clear, and no tenderness was found. The mass could move up and down with swallowing. There is no jugular vein distension, and the trachea is centered. No enlarged lymph node was found through palpation. No other signs were found in the physical examination. The patient denied symptoms such as fever, palpitations, shaky hands, and irascibility. There were no abnormalities in defecation and urine. There was no significant change in weight over the past 3 months. The patient underwent a cholecystectomy 10 years ago. She had a history of asthma for a year. Intermittent oral and inhaled drug therapy were used for asthma. The patient has no familial-hereditary disease, and no similar diseases were diagnosed in her family.

### Accessory examination before surgery

**Figure 2:**
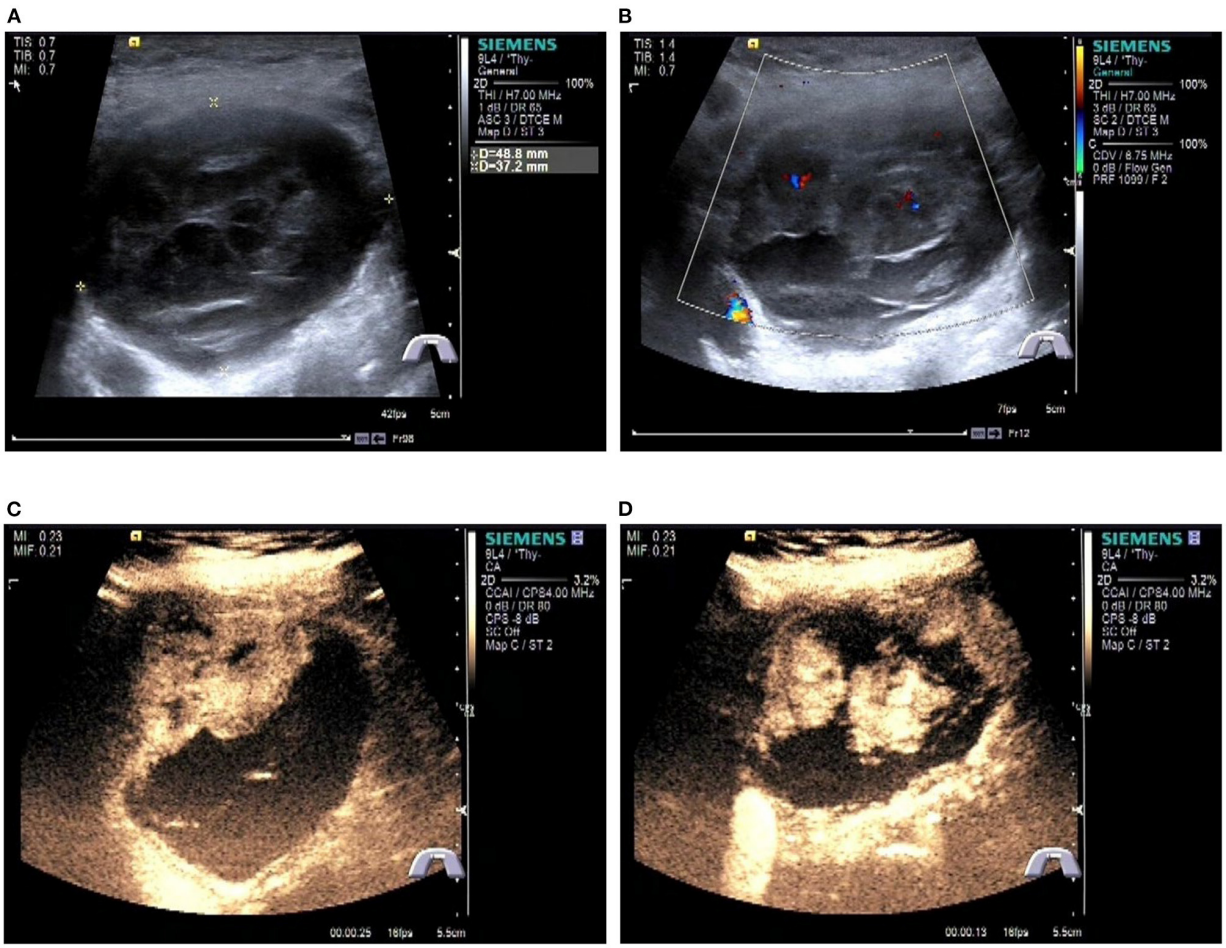
Preoperative contrast-enhanced ultrasonography of the patient. **(A)** Gray scale ultrasonography of the mass. **(B)** Color Doppler imaging of the mass. **(C, D)** Contrast-enhanced ultrasonography of the tumor.

**Figure 3:**
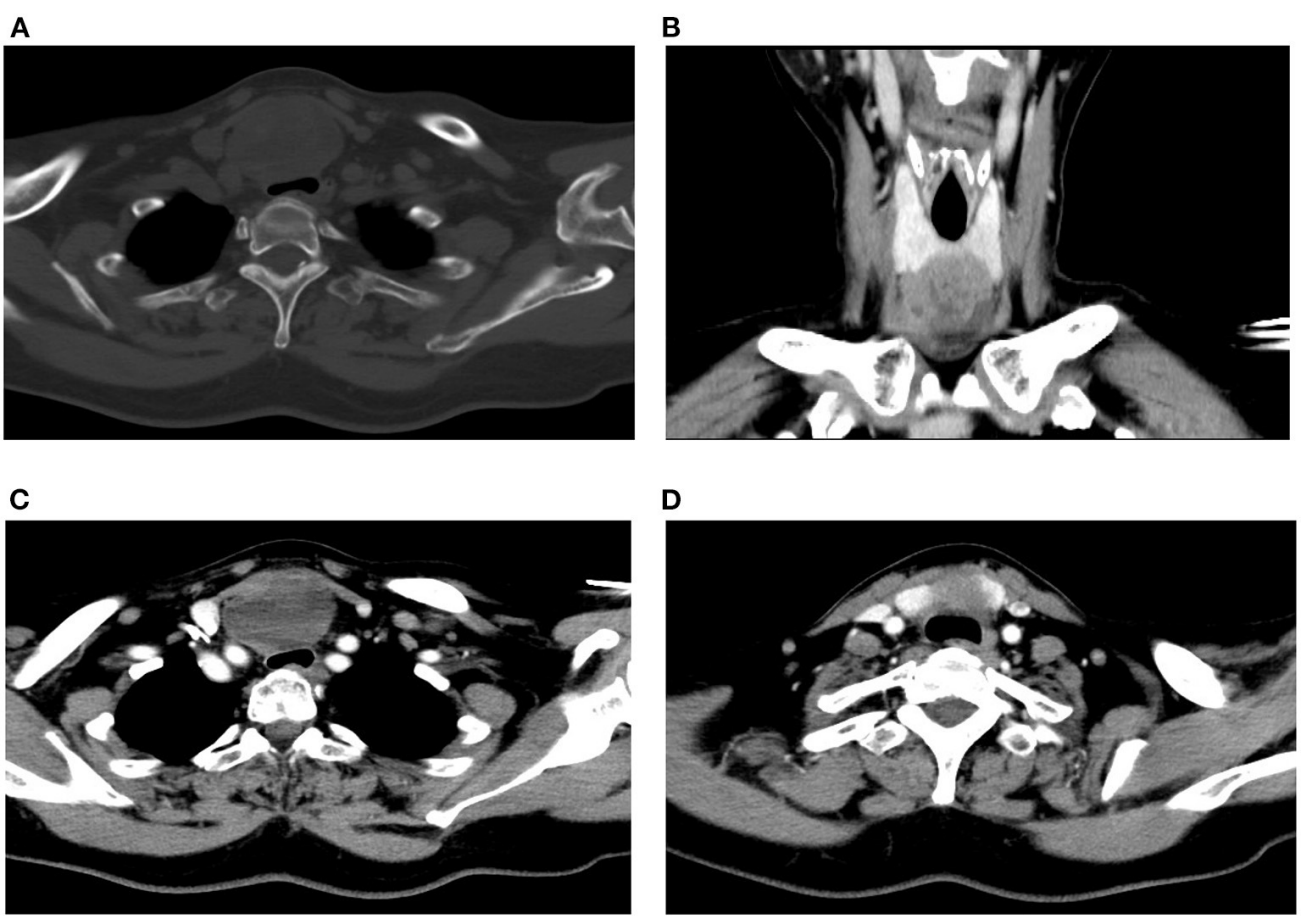
Preoperative CT scan of the patient. **(A)** Plain CT of the neck (horizontal plane). **(B)** CT scan of the neck (coronal plane). **(C, D)** Contrast-enhanced CT of the neck (horizontal plane).

A Blood test showed no obvious abnormalities. The level of thyroid hormone and calcitonin is in the normal range. No abnormalities were found in the laryngoscopy, chest X-ray, and chest CT. Contrast-enhanced ultrasonography showed a heterogeneous echoic, cystic-solid mixed mass ~48.8 × 37.2 mm in the isthmus of the thyroid gland. The mass is with clear boundary, regular shape. In total, 0.6 ml perflubutane microspheres were injected through the elbow intravenous for CEUS. The isthmus nodule was mostly equally enhanced and partially no enhancement. The peripheral nodule showed annular enhancement. No obvious enlarged lymph nodes were observed in the cervical region. The mass was classified as TI-RADS 3 (Figure 2). CT scan showed a mass lesion in or below the isthmus of the thyroid gland. The mass was heterogeneous density. There are microcalcifications and liquefaction necrosis areas inside the mass. The average CT value is 28 HU. The edge of the mass is mostly clear. The contrast-enhanced CT demonstrated heterogeneous enhancement. The degree of enhancement is lower than that of the normal thyroid gland. The average CT value is 58 HU. There are multiple small lymph nodes in the I-II area of the cervical region. The CT could not establish a confirmed diagnosis but a low-grade tumor could not be ruled out (Figure 3).

### Therapy

The patient was intended for partial thyroidectomy. We made a transverse arc-shaped incision in the suprasternal notch, dissociated the subcutaneous tissue, and opened the interval between the bilateral strap muscles in the neck to expose the thyroid mass. The mass was located in the isthmus of the thyroid gland and extended to the back of the sternum. The mass was cystic-solid mixed. The cyst wall was closely adhered to the trachea. Intraoperatively, the mass was difficult to be mobilized and led to a capsular rupture and an effusion of necrotic, hemorrhagic, and gelatinous liquid. When the mass was cut open, chocolate-like contents could be seen in the capsule. The solid section was fish-like, gray-red, and gray-yellow, and hard calcification was visible in some areas. Frozen section pathology showed soft tissue sarcoma, after which we choose to carry out total thyroidectomy and central cervical lymph nodes dissection.

### Histopathology

**Figure 4:**
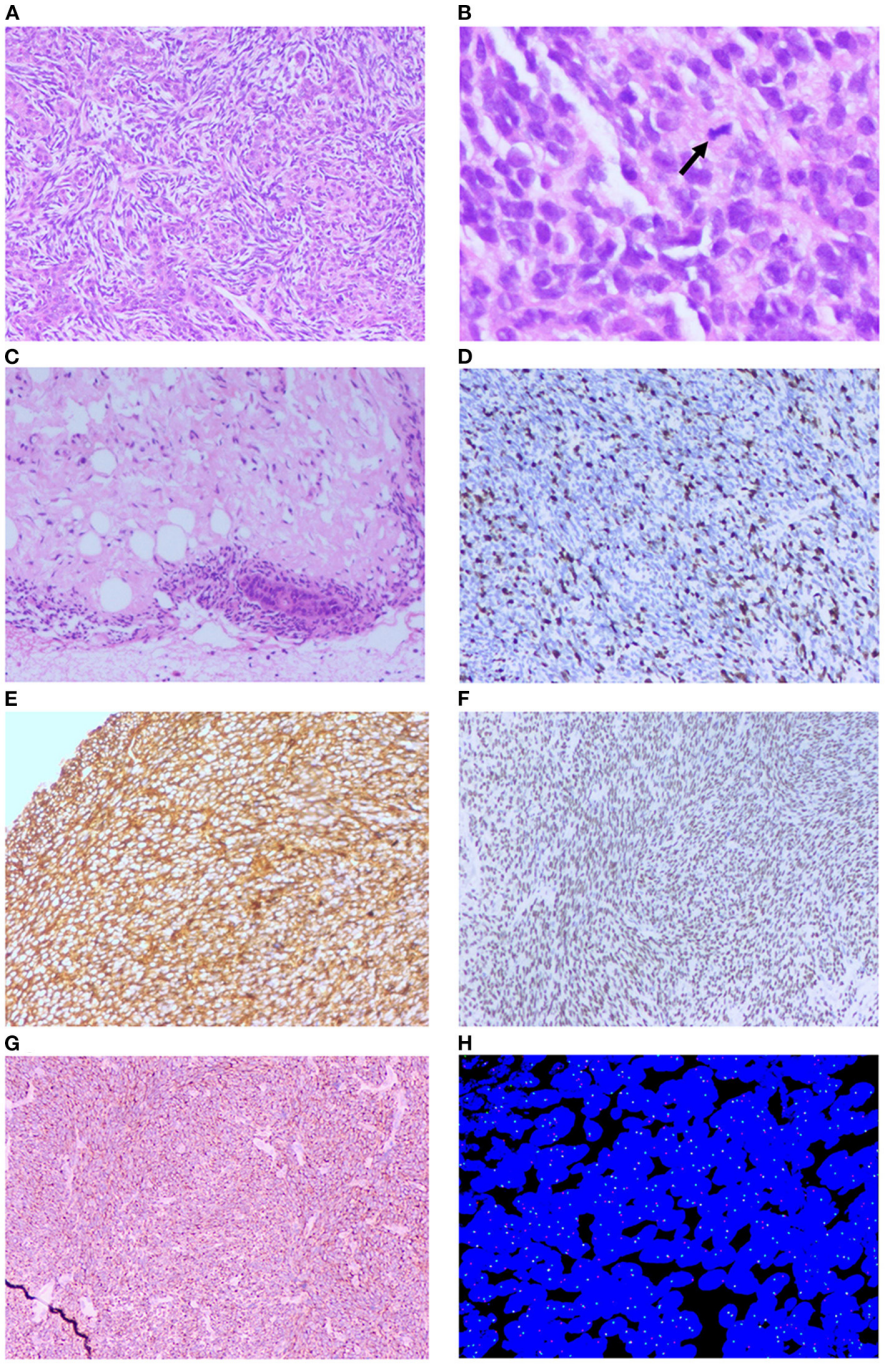
Histopathology examination and FISH of the patient. **(A)** Microscopic images showing biphasic tumor (H&E × 100). **(B)** Microscopic images showing pathological mitotic (Black arrow, H&E × 400). **(C)** Microscopic images showing the tumor invasion to adipose tissue (H&E × 100). **(D)** Immunohistochemistry positive to Ki-67 (H&E × 100). **(E)** Immunohistochemistry positive to Vim (H&E × 100). **(F)** Immunohistochemistry positive to TLE1 (H&E × 100). **(G)** Immunohistochemistry positive to Bcl-2 (H&E × 100). **(H)** Fluorescence *in situ* hybridization (FISH) break-apart probe confirmed SS18 gene rearrangement (split red and green signal).

Histopathology showed that the thyroid isthmus mass was a mesenchymal tissue-derived spindle cell malignant tumor, with epithelioid differentiation in the focal area. The nucleus was atypical. Lots of mitotic figures could be seen. Invasion of surrounding soft tissues was observed. There are no obvious masses in the left and right lobes of the thyroid gland. There was no metastasis in the central cervical lymph nodes (0/6) (Figure 4).

### Immunohistochemistry

CK(-), EMA(-), CK7(-), CK19(-), CD99(-), Bcl-2(+), WT-1(-), TLE1(+), Ki-67(50%), P53(30%+), Vim(+), Syn(focally +), S100(-), SMA(-), MyoD1(-), STAT6(-), and SOX-10(-) (Figure 4).

### Fluorescence *in situ* hybridization (FISH)

Molecular analysis, performed on the formalin-fixed paraffin-embedded tissue by fluorescent *in situ* hybridization (FISH), showed an SS18 (SYT) gene rearrangement, highly consistent with the diagnosis of a PSST (Figure 4).

**Pathology Stage:** pT2N0M0. Stage IIA

### Postoperative adjuvant therapy and follow-up

After the initial surgery, chest CT, abdominal CT, MRI of the head, and whole-body bone scan were performed for evaluation. No obvious local and distant recurrence or metastases was found. After evaluation, this patient received chemotherapy (a combination of ifosfamide 3,000 mg and epirubicin 120 mg). The patient tolerated chemotherapy well. The patient received five-cycle chemotherapy. Postoperative adjuvant radiation therapy was recommended by the oncologist, but the patient refused for personal reasons. After 9 months of the surgery, no remarkable recurrence and metastases were detected.

## Discussion

In this article, we reported the 16th PSST case globally. Although the mass of this patient was mostly composed of spindle cells, a lack of normal thyroid follicular epithelial cells, the boundary between the mass and the surrounding thyroid tissue was unclear, and a part of the normal thyroid tissue was replaced by the synovial sarcoma. In synovial sarcoma of the head and neck regions originating from other structures, the main tumor foci are usually situated around their local regions instead of the thyroid gland. Therefore, according to a definition in a previous study, this mass was identified to be primary thyroid synovial sarcoma (5). When summarizing the clinical features of this case, we could find that the course of the disease was only 20 days, and the mass has some similarities with the benign thyroid nodule (including clear boundary, regular shape, and annular enhancement on ultrasound). These characteristics may lead to a missed diagnosis of malignancy in preoperative evaluation. However, when trying for total excision, we found that the mass closely adhered to the trachea, which is different from a typical benign thyroid tumor. Therefore, we choose to perform frozen section pathology intraoperatively. The intraoperative frozen section pathology assisted the surgeon to make clinical decisions and helped the patient to avoid reoperation. According to the research by Maduekwe et al. synovial sarcoma was thought to have a predilection for metastases to lymph nodes, necessitating the need for possible sentinel node biopsy (15, 16). Therefore, when we encounter rapidly growing thyroid nodules, we should be careful in perioperative assessment and perform intraoperative frozen section pathology when necessary.

For further investigation, we searched the PSST case in PubMed and Web of Science. A total of 15 cases were reported previously. We summarized the clinical characteristics of the 15 cases in Table 1. In most PSST cases, the course of the disease is < 1 year and progressing rapidly. In cases with detailed follow-up, most PSST patients presented recurrence within 2 years. A total of eight cases have detailed survival data. Among them, six patients have fatal events. The longest survival time in these patients is 126 months (in a study by Carina Owen) and four patients died within 3.5 years. The 5-year survival rate is consistent with a previous study (14). Generally, PSST showed rapid disease progression and relatively poor prognosis. Regarding the preoperative evaluation of PSST, the tumor is usually cystic-solid mixed, larger than 5 cm, and presented symptoms of neck compression. In eight cases, fine needle aspiration (FNA) was performed for diagnosis. However, only one case has been suspected to be a malignant spindle cell tumor. Most patients were diagnosed with MTC or undifferentiated thyroid carcinoma by FNA, and some patients could not be diagnosed by FNA. These data indicated that the cytopathological diagnosis of PSST is challenging. Although Aisner et al. proposed that FNA is important for diagnosing soft tissue synovial sarcoma, establishing the diagnosis of PSST is difficult due to its rarity (17). We should be careful in clinical practice in order to reduce misdiagnose rate of this rare disease even when FNA indicated a benign thyroid tumor, especially in a rapidly growing, cystic-solid mixed thyroid mass with neck compression symptoms.

**Table 1:** Clinical characteristics of PSST cases reported previously.

**No.**	**Author**	**Year**	**Age/gender**	**Chief complaint**	**Course**	**Tumor size**	**Ultrasound**	**CT**	**Initial diagnosis**	**FNA**	**Surgery**	**Histopathology and metastasis**	**Immunohistochemistry**	**Gene analysis t (X; 18) (p11; q11) & Tech **	**Subtype**	**Postoperative adjuvant therapy**	**Recurrence**	**Death**	**Last follow-up (months after surgery)**
1	Inako Kikuchi	2002	60/Male	Hoarseness	1 year	6.8*6.5 cm	Not recorded	Cervical tumor to be accompanied by destruction of the thyroid and cricoid cartilage	Thyroid medullary carcinoma (by FNA)	Thyroid medullary carcinoma	Total thyroidectomy with partial resection of the thyroid and cricoid cartilage, partial resection of the cervical esophagus and dissection of the lymph nodes around the thyroid gland	Mostly long spindle cells	vimentin (+), bcl-2 (+), epithelial membrane antigen (+), cytokeratin (+), Leu-7(+), thyroglobin (-), triiodothyronine (-), thyroxin (-)	Chimera SYT-SSX gene transcripts (by RT-PCR)	Monophasic	Not recorded	Multiple pulmonary metastases and local tumor recurrence (18 months after surgery)	32 months after the surgery	32 months
2	Ki-Seok Jang	2007	15/Male	Palpable neck mass	4 months	6*5*5 cm	Not recorded	A relatively well-demarcated solid mass at the superior and lateral portion of the left thyroid gland	A benign follicular lesion (by FNA)	Moderate cellularity with a predominance of bipolar spindle-shaped cells	Total thyroidectomy and left cervical lymph node dissection	An admixture of spindle and epithelial cell components in almost equal proportions	Cytokeratin (+), vimentin (+), epithelial membrane antigen (+), CD 99 (+), muscle specific actin (-), smooth muscle actin (-), S-100(-), carcinoembryonic antigen (-), synaptophysin (-), chromogranin (-), thyroglobulin (-), and thyroid transcription factor-1(-)	SYT-SSX fusion gene transcript was identified (by RT-PCR)	Biphasic	Not recorded	Not recorded	Not recorded	Not recorded
3	Chang Hwan Ryu	2011	72/Female	Neck mass, hoarseness and dysphagia	3 months	6*5*4.5 cm	A 6*5cm-sized, heterogeneously hypoechoic nodule with internal calcification in the left lobe of the thyroid gland	A large, low attenuating mass replacing the left thyroid gland extending to the level of the hyoid bone superiorly and superior mediastinum inferiorly with possible invasion to the trachea	Cystic change (by FNA)	Cystic change	Total thyroidectomy with tracheal fenestration	Fascicles and sheets of dense, uniform, relatively small ovoid neoplastic cells	CD 99(+), cytokeratin (-), desmin (-), S-100(-), CD 31(-), CD 34(-), epithelial membrane antigen (EMA) (-)	SYT/SSX fusion gene transcript (+)	Monophasic	Concurrent chemoradiation was planned but not performed	Not recorded	2 months after surgery (due to unknown causes)	2 months
4	Ali Ghafouri	2012	44/Female	Neck mass and dyspnea	4 months	17*14*6cm	Not recorded	A large tumor with tracheal deviation	Unknown	Not recorded	Mass biopsy and further surgery (not recorded in detail)	Spindle cell tumor with pleomorphism and necrosis	Thyro (-), TTF1(-), EMA (-), vimentin (+), EGFR(+)	Not recorded	Monophasic	Not recorded	Not recorded	Not recorded	Not recorded
5	Laurys Boudin	2014	55/Male	Palpable neck mass and dysphagia	1 month	Over 12cm in diameter	A 7cm mass of the left thyroid lobe which was heterogeneous, hypervascularized, and mixed (solid and liquid)	Not recorded	Anaplastic thyroid carcinoma	/	Total thyroidectomy	A dense cellular proliferation composed of ovoid-shaped to frankly spindle-shaped cells	EMA (+), pan-cytokeratin (-), desmin (-), H-caldesmon (-), PS100 (-), HMB-45 (-), MDM2(-)	SYT gene rearrangement (by FISH)	Monophasic	Combination of doxorubicin and ifosfamide	A 4cm cervical mass was found (15 days after surgery)	Not recorded	Not recorded after the revision surgery
6	RONG-LIANG SHI	2016	31/Male	Neck mass without symptom	1 month	5*2*2 cm	A large mass with an area of 5*2*2 cm in the right thyroid lobe, which was heterogeneous, hypervascular and solid	Not recorded	Poorly differentiated tumor	/	Total thyroidectomy	The tumor contained two components spindle cells and glandular structures	B-cell lymphoma-2(+), cytokeratin (+)	Not recorded	Biphasic	Adjuvant chemotherapy containing ifosfamide and doxorubicin was administered every 3 weeks for four cycles. A total of 50 Gy of adjuvant radiotherapy was applied.	A patchy LN measuring 2 cm in diameter in the right upper neck (2 years after initial surgery)	Not recorded	34 months
7	Chang-Soo Park	2017	47/Female	Thyroid mass detected on regular health check up	Not recorded	7.8mm in diameter	7.8mm sized anechoic lesion suspected of malignancy was found in the isthmus of right lobe	Not recorded	Unknown	Large quantity of either single or clustered ovoid to spindle cells	Total thyroidectomy	The tumor was composed of ovoid to spindle tumor cells, forming fascicles with an interlacing arrangement	epithelial membrane antigen (+), CK (+), vimentin (+), transduction-like enhancing protein 1(+), neuroendocrine markers (-), CD56(-), synaptophysin (-), chromogranin (-), calcitonin (-), CD34(-), thyroid transcription factor-1(-), S-100(-), c-kit (-)	Translocation (X; 18) (p11;q11) (confirmed by FISH)	Monophasic	Not recorded	No recurrence or metastasis was detected	Alive until 3 years after surgery	36 months
8	Diksha Yadav	2018	12/Male	A gradually progressive neck mass associated with dyspnea, dysphagia and voice change	1.5 years	12*11*7cm	Not recorded	A 9.1*6.9*6.1cm heterogeneously enhancing lesion involving left lobe of thyroid and adherent to the surrounding structures	Malignant spindle cell tumor or MTC	Malignant spindle cell tumor, with a possibility of non-secretory MTC was rendered	Total thyroidectomy	Spindle cell tumor with small nests of polygonal cells with focal glandular pattern	CK7 (+), CK19 (+), EMA (+), BCL2 (+), CD99 (+)	Translocation (X;18) (p11.2: q11.2) (confirmed by FISH)	Biphasic	Not recorded	Not recorded	7 months after surgery	7 months
9	Carina Owen	2018	37/Male	Initial symptoms were not recorded. Chest pain and haemoptysis were presented when recurrence occurs.	Not recorded	Not recorded	Not recorded	Not recorded	Unclear	Not recorded	Total thyroidectomy and De-bulking surgery	Histopathology of primary site was not recorded. Histopathology of metastatic lesion showed synovial sarcoma, and showed a relatively circumscribed cellular neoplasm, which entrapped lung parenchyma and was composed of elongated slender spindle cells with minimal pleomorphism and mitotic activity.	TLE1(+), focal bcl-2(+), cytokeratins (-), MNF116(-), AE1/AE3(-), epithelial membrane antigen (-), S100 protein (-), CD34(-), TTF-1(-), CD99(-), WT1(-), SMA (-), desmin (-), MUC4(-), CD31(-), CD117(-), DOG1(-), EBER (-), STAT6(-), HHV8(-), calretinin(-)	SS18 gene rearrangement (by FISH)	Suspected Monophasic (not described in detail)	Postoperative radiotherapy; Combination chemotherapy of doxorubicin and ifosfamide; Second-line chemotherapy consisting of an infusional ifosfamide schedule; 4 cycles of trabectedin	8 years after initial surgery	126 months after initial surgery	126 months after initial surgery
10	Carina Owen	2018	47/Male	An asymptomatic left-sided thyroid nodule	Not recorded	Not recorded	Not recorded	A CT-positron emission tomography (PET) scan showed no distant sites of metastatic disease.	Unknown	A core biopsy was performed, but the diagnosis was not established	Total thyroidectomy and bilateral lymph node dissection. Following chemotherapy, he underwent resection of the two right lung metastases followed by left thoracotomy and resection of a left upper lobe metastasis.	Fascicles of minimally atypical spindle cells with intermixed islands of epithelioid cells. No lymph nodes metastasis was found.	CD56(+), TLE (+), Cytokertins(-), EMA(-)	SS18-SSX2 fusion (by RT-PCR)	Biphasic	Combination therapy with doxorubicin and ifosfamide and post-operative radiation to the left upper lung	Pulmonary metastatic presented two years after initial surgery	55 months after initial surgery	55 months
11	Carina Owen	2018	42/Male	Neck mass	Approximately 5 years	3.5cm	Not recorded	Not recorded	Paraganglioma	Not performed	Tumor excision (Not described detailed)	Fascicles of minimally to mildly spindle cells with elongated or ovoid nuclei, indistinct nucleoli and scanty cytoplasm, and a mitotic index of up to 16/10 hpf.	EMA (+), bcl-2(+), S100(+), MNF116(-), CAM5.2(-), CK903(-), CD99(-), CD34(-), CD117(-), SMA(-), HMB45(-), CD31(-), desmin(-) and neurofilament(-)	SS18 gene rearrangement (confirmed by FISH)	Monophasic	Lung metastases excised and conventional chemotherapy	Multiple lung metastases were found 1years after initial surgery	Not recorded	72 months (no further follow-up was performed)
12	Carina Owen	2018	36/Male	A lump on neck (with shortness of breath and difficulty in swallowing)	1 month	5.7*7.5*9cm	Not recorded	A large mass in the right lobe of the thyroid gland (5.7 × 7.5 cm in the axal plane and craniocaudal, up to 9 cm) as well as numerous lung nodules consistent with metastases	Synovial sarcoma	Not recorded in detail	Not resectable	Not recorded in detail	Not recorded in detail	Translocation involving the SS18 gene at 18 q 11.2.	Not recorded	Combination of doxorubicin and ifosfamide. Radiation and an ATR inhibitor were used in followed therapy.	Not recorded in detail	Not recorded	Not applicable
13	Carina Owen	2018	30/Female	A tumor in the right thyroid lobe which involved adjacent structures in the neck	Not recorded	Not recorded	Not recorded	Not recorded	Poorly differentiated synovial sarcoma	Not recorded	Not recorded	A cellular tumor, composed of sheets of relatively monotonous, mildly atypical rounded to ovoid cells with vesicular nuclei and scanty cytoplasm.	CD56(+), Cytokertins (focally +), EMA (focally +)	Not recorded	Not recorded	Not recorded	Not recorded	Not recorded	Not recorded
14	Dilaver Demirel	2020	26/Male	A solid, lobulated mass in the left thyroid lobe	Not recorded	Primary tumor: 4.5cm; Recurrent neck tumor: 5 × 2.5 × 2cm	Not recorded	CT of initial tumor was not scanned. MRI of recurrent tumor shows 6.4 × 4.5 × 3.4cm mass, with necrosis and suspicious invasion of the trachea	Papillary carcinoma (by FNA); Spindle epithelial tumor with thymus-like differentiation (Histopathology of first surgery)	Papillary carcinoma	Total thyroidectomy and lymph node dissection for primary site; Partial laryngectomy for recurrence	Primary tumor: Spindle epithelial tumor with thymus-like differentiation with no evidence of any nodal (0/8) or distant metastasis; Recurrent tumor: diagnosed with synovial sarcoma by	Pan-CK(+), vim(+), TLE-1(+), Bcl-2(+), CD99(+), FLI-1(+), CD56(+), EMA(+), CK-7(+), calponin(+), Ki-67 (50%+); Thyr(-), calc(-), chrom(-), syn(-), CEA(-), CK 8/18(-), HMWCK (34 beta-), P63(-), SMA(-), TTF1(-), CK-20(-), CD34(-), S100(-), des(-)	SYT-SSX1 fusion transcript was verified by FISH and RT-PCR	Biphasic	Radioactive iodine (PT)/ chemotherapy and targeted therapy	Local recurrence: 24 months, lungs, thoracic and lumbar vertebrae, iliac bone: 32 months	41 months after surgery	41 months after surgery
15	Seyed-ahmad Seyed-alagheband	2021	43/Female	Dysphagia and dyspnea	7 months	10*8*4 cm	Hypervascularized and mixed (solid and liquid) thyroid mass	A heterogeneous thyroid mass with a 90*45*65 mm thyroid nodule with internal necrotic view	Undifferentiated cell carcinoma of thyroid gland	Undifferentiated cell carcinoma of thyroid gland	Total thyroidectomy	Fascicles and sheets of monophasic spindle cell growth pattern with oval nuclei	EMA (+), CK 7(+), CK 19(+), BCL-2(+), TLE1(+), S-100(-), CD99(-), CD31(-), CD34(-), Ki-67(5%+)	SYT-SSX fusion gene transcript was identified (by RT-PCR)	Monophasic	6 cycles of doxorubicin and ifosfamide and radiotherapy	No recurrence or metastasis was detected	Alive	Not recorded

PSST was usually misdiagnosed with MTC, undifferentiated/anaplastic thyroid carcinoma, or SETTLE. In this part, we would discuss the differential diagnosis of PSST. For PSST, the main differential diagnosis is MTC. An elevated calcitonin level is typical in MTC patients. In a microscopic vision, the MTC tissue usually consists of lympho/plasmacytoid cells, atypical cells, or spindle cells. However, a pure spindle cell pattern MTC is uncommon. Giant cells or multinucleated cells could be seen. The MTC tumor cells are generally positive for calcitonin, thyroglobulin, and neuroendocrine markers (18). SETTLE is simple for spindle epithelioid tumors with thymus-like differentiation. SETTLE is common in children and young adults. The mass is slow-growing. The incidence in male is higher than in female. Similar to PSST, the tumor consists of spindle cells with a variable epithelial component. In SETTLE, the mitotic rate is usually low and necrosis is relatively rare. The spindle cells are positive for high-molecular-weight keratin and vimentin (19). Occasionally, PSST would be misdiagnosed as undifferentiated/anaplastic thyroid carcinoma (UTC/ATC). UTC/ATC is usually presented as a rapidly enlarging thyroid mass. It is more common in elderly patients. The tumor is composed of spindle cells with ample cytoplasm, coarse chromatin, frequent mitoses, giant cells, and neutrophils. UTC/ATC is positive for Pax-8 and has p53 mutations (20).

Until now, there is no standard and ideal therapeutic regimen for PSST. Surgical resection remains the mainstay of management for localized synovial sarcoma with or without radiation (11). When the tumor invades important structures and cannot be completely removed, postoperative adjuvant chemoradiotherapy is generally recommended. Some retrospective studies have reported an improvement in local control and disease-free survival conferred by radiotherapy (21–23). A combination of doxorubicin and ifosfamide was considered to be the recommended chemotherapeutic regimen when the primary tumor could not be resected completely in most cases. While PSST did not show an obvious genetic predisposition, the consanguinity of the patient should raise their awareness and perform a regular physical examination.

### Limitation

Although we tried our best to document this clinical case report in detail, there are still some limitations. First, because the tumor, in this case, was suspected to be benign in the preoperative examination, we did not perform screening for systemic metastases before surgery, but relevant examinations including chest CT, abdominal CT, and MRI of the head and whole-body bone scan were performed before and during chemotherapy. Second, due to capsular rupture and necrotic effusion during the operation, we are pitiful for failing to take photos of the tumor. The necrotic effusion might lead to tumor local implantation metastasis, making postoperative adjuvant therapy necessary and important. Last but not the least, despite the follow-up of the case being less than 1 year, complete treatment for PSST in this patient has been completed, and no recurrence and metastasis have been detected in 9 months postoperatively. We will continue to focus on the case. We will update this case report and literature review if recurrence or death occurs in the current case.

## Established facts

1. Synovial sarcoma is a subtype of soft tissue sarcoma, which is extremely rare in the thyroid gland. PSST showed rapid disease progression and a relatively poor prognosis. However, diagnosis and therapy are challenging.

2. Complete surgical excision and postoperative adjuvant chemotherapy are usually the typical therapeutic regimen for PSST.

## Novel insights

1. PSST is difficult to be diagnosed by preoperative evaluation, even FNA. PSST was occasionally misdiagnosed as a benign thyroid tumor. Surgeons should raise their awareness when encountering rapidly growing, cystic-solid mixed thyroid mass with neck compression symptoms.

2. The initial surgery of PSST is of vital importance. Surgeons should avoid capsular rupture and effusion of necrotic during surgery. Intraoperative frozen section pathology should be performed when necessary. Postoperative adjuvant chemotherapy should be scheduled for reduced recurrence and prolonged survival time.

## Data availability statement

The original contributions presented in the study are included in the article/supplementary material, further inquiries can be directed to the corresponding authors.

## Ethics statement

Ethical review and approval was not required for the study on human participants in accordance with the local legislation and institutional requirements. The patients/participants provided their written informed consent to participate in this study. Written informed consent was obtained from patient for the publication.

## Author contributions

JH, YG, FY, and CR performed the surgery. CR, ZC, QJ, and YL collected the data from the patient. CR, XL, and SL drafted the first manuscript. YG and FY revised the manuscript. All authors read and approved the final manuscript.
